# The Relationship Between Technology Use and Problem Technology Use and Potential Psychosocial Mechanisms: Population-Based Telephone Survey in Community Adults During COVID-19

**DOI:** 10.3389/fpsyg.2021.696271

**Published:** 2021-08-09

**Authors:** Xue Yang, Benjamin H. K. Yip, Eric K. P. Lee, Dexing Zhang, Samuel Y. S. Wong

**Affiliations:** ^1^Jockey Club School of Public Health and Primary Care, Faculty of Medicine, The Chinese University of Hong Kong, Shatin, Hong Kong; ^2^The Chinese University of Hong Kong Shenzhen Research Institute, Shenzhen, China

**Keywords:** digital addiction, COVID-19, post-traumatic stress disorder, boredom, loneliness

## Abstract

**Background:** Although digital technology enables people to stay connected during COVID-19, protracted periods of isolation, crisis-induced stress, and technology-based activity may intensify problem technology use (PTU), such as social media addiction (SMA) and Internet gaming disorder (IGD).

**Objective:** This study aimed to characterize the patterns and levels of SMA and IGD during COVID-19 in the general population of Hong Kong. We also tested the associations between prolonged use of social media/Internet games and SMA/IGD and the mediation effects of psychosocial statuses (i.e., loneliness, boredom, and post-traumatic stress) on these associations.

**Methods:** A population-based random telephone survey was conducted in community adults in May 2020; 658 social media users and 177 Internet gamers were identified. A structured questionnaire, including the Bergen Social Media Addiction Scale, the diagnostic and statistical manual of mental disorder IGD Symptoms Checklist, the Post-Traumatic Stress Disorder Scale, Multidimensional State Boredom Scale, and the De Jong Gierveld Loneliness Scale, was used. Time spent on social media and Internet games during and before COVID-19 was also asked.

**Results:** There were 66.2–81.8% increases in time use of social media or Internet games during COVID-19 compared to pre-COVID-19 self-reported information of the participants. The estimated IGD prevalence rate in the gamers based on the sample weighted to the age distribution and gender ratio of the Hong Kong population was 9.7%, higher than that of pre-COVID-19 research. Age, marital status, education levels, time use of social media, COVID-19-related post-traumatic stress, boredom, and emotional loneliness were significantly associated with SMA, while time spent on Internet games, boredom, and emotional loneliness was significantly associated with IGD. Boredom positively mediated the associations between time spent on social media/Internet games and SMA/IGD, whereas social loneliness negatively mediated the association between time spent on social media and SMA.

**Conclusion:** These findings highlight the concern of prolonged use of digital platforms during COVID-19 and its role as a “double-edged sword” for psychosocial wellbeing and behavioral health during COVID-19. It also highlights a need to monitor and prevent PTU in the general public. The observed psychosocial mechanisms are modifiable and can inform the design of evidence-based prevention programs for PTU.

## Introduction

The COVID-19 pandemic and the relevant control measures (e.g., lockdown, quarantine, social distancing, and home confinement) have greatly increased usage of social media and online gaming (King et al., 2020). According to Verizon, an American telecommunications company, there was a 75% surge in gaming data usage in the United States amid the initial implementation of the stay-at-home directives (Clifford, 2020). Sensor Tower, a market intelligence platform, also reported a 19% increase in mobile game download in Europe under strict lockdown measures in March (Chapple, 2020). Facebook reported an 11% increase in daily active users and that voice and video calls on Facebook Messenger and WhatsApp nearly doubled in regions most affected by COVID-19 (Facebook, 2020; Schultz, 2020). Empirical research based on self-report of the participants also revealed a similar trend (Cellini et al., 2020). For example, a study in China found that 16.6% of the participants spent longer time on the Internet during the pandemic than pre-pandemic (Sun et al., 2020). Górnicka et al. conducted a cross-sectional online survey in Polish adults and found that 49% of the participants reported an increase in screen time during the pandemic compared to pre-pandemic (Górnicka et al., 2020).

Prolonged technology use and long screen time may intensify problem technology use (PTU; i.e., problematic or addictive use of technology), such as social media addiction (SMA) and Internet gaming disorder (IGD; King et al., 2020). Social media addiction occurs when the condition of social networking sites (SNS) use fulfills six criteria (i.e., salience, mood modification, tolerance, withdrawal symptoms, conflict, and relapse; Griffiths et al., 2014). Internet gaming disorder refers to the “persistent and recurrent use of the Internet to engage in games, often with other players, leading to clinically significant impairment or distress” (American Psychiatric Association, 2013). Problem technology use has broadly adverse and profound effects on mental and physical health, including sleep disorders, depression, anxiety, suicidal ideation, neck and elbow pain, and obesity (Weinstein, 2010; Achab et al., 2011; Griffiths et al., 2012; AlZarea and Patil, 2015; Keles et al., 2020). Particularly, IGD was listed in the Diagnostic and Statistical Manual of Mental Disorder (DSM-5) as a condition for further research (American Psychiatric Association, 2013) and was defined as a mental disorder in the eleventh edition of the International Classification of Diseases (World Health Organization, 2018). The expert consensus on the need of monitoring and preventing SMA and IGD during COVID-19 has been highlighted by a couple of commentaries (Górnicka et al., 2020; Ko and Yen, 2020). We have not identified any population-based study that invested the level/prevalence of PTU during COVID-19, although several empirical studies using convenience samples were conducted and most of them focused on students/young populations (e.g., college students; Boursier et al., 2020; Chen et al., 2020; Brailovskaia and Margraf, 2021; Elhai et al., 2021; Paschke et al., 2021; Zhao and Zhou, 2021). Two studies tested the relationship between technology use and PTU during COVID-19 (Shrestha et al., 2020; Chen et al., 2021). Both focused on students and reported positive relationships between time spent on gaming/SNS/smartphone and problematic gaming/SNS use/smartphone use.

During this special period, technology use may have both harmful and beneficial effects on individuals’ psychosocial statuses, and such statuses may further play as mediators to explain how technology use may increase or reduce PTU. First, prolonged use of digital technology, such as SNS, may be a significant source of stress (e.g., post-traumatic stress) and negative emotions (e.g., boredom) because it may increase repeated exposure to the stressful event and negative information related to the crisis and facilitate the spread and contagion of negative emotions (Fung et al., 2014; Kramer et al., 2014; Depoux et al., 2020). In turn, increased stress and negative emotions may lead to PTU. For example, an online survey in adults in Canada and United States reported that health anxiety and fear of missing out were positively correlated with problematic smartphone use and gaming disorder (Elhai et al., 2021). Another study reported positive associations between burden by COVID-19/anxiety and addictive social media use (Brailovskaia and Margraf, 2021). Therefore, technology use may increase PTU through increased post-traumatic stress and boredom during COVID-19. We have not identified any empirical research on the roles of post-traumatic stress or boredom in relating to technology use and PTU.

On the other hand, it is expected that technology use may reduce loneliness by maintaining one’s social network and ameliorating the negative impact of prolonged isolation during COVID-19 (Liu and Kim, 2011; Li and Wang, 2020; Zhou et al., 2020; Gong et al., 2021). In turn, decreased loneliness may protect one from negative coping behaviors and PTU. Loneliness is a well-documented risk factor of PTU in the previous non-COVID-19 studies (Seay and Kraut, 2007; T’ng et al., 2020). One study in Italian adults conducted during COVID-19 also reported a significant positive correlation between loneliness and SMA (Boursier et al., 2020). Thus, technology use may reduce PTU through reduced loneliness during COVID-19 (Boursier et al., 2020).

These mediation effects may be theoretically supported by the Conservation of Resources theory (COR; Hobfoll, 1989). According to the COR (Hobfoll, 1989), technology use may enhance or deteriorate ones’ wellbeing through facilitating their gain or loss of resources. These resources, such as personal resources (e.g., a psychological state, information, and health condition) and interpersonal resources (e.g., social support and social network), can influence how individuals appraise and cope with stress, and thus changes in these resources affect mental and behavioral disorders (Hobfoll, 1989). It is because these psychosocial statuses are important resources of stress coping; increases in these negative statuses may limit one’s stress coping capacity, and increase the likelihood of maladaptive coping (e.g., avoidance and escape), thus leading to excessive/maladaptive use of digital technology and PTU (e.g., Lemmens et al., 2009; Zhou and Leung, 2012; van Rooij et al., 2014; Kardefelt-Winther, 2014b; Cheng et al., 2018). This theory was originally developed to explain individuals’ mental health in traumatic events, and it has been applied to explain the role of digital technology use in mental health problems, such as depression and anxiety (Lagana and Garcia, 2013; Feldman et al., 2015; Montani et al., 2018; van der Velden et al., 2019). In this light, digital technology use during COVID-19 may affect one’s psychosocial resources (e.g., post-traumatic stress, boredom, and loneliness) which in turn lead to or protect one from PTU and other mental disorders. To our knowledge, no study has tested such mediation models. This study would fill this research gap.

### The Present Study

This study aims to test two research questions. First, whether time spent on technology use (social media/SNS and Internet games) would increase PTU (SMA and IGD) during COVID-19. Second, whether this relationship would be mediated by three types of psychosocial statuses (i.e., post-trauma stress, boredom, and loneliness). It is hypothesized that time spent on technology use would be positively associated with PTU during COVID-19. Also, it is hypothesized that time spent on technology use would be positively associated with post-traumatic stress and boredom, and negatively associated with loneliness; in turn, post-traumatic stress, boredom, and loneliness would be positively associated with the levels of PTU. In other words, these psychosocial statuses would explain the relationships between time spent on technology use and PTU.

**Figure 1:**
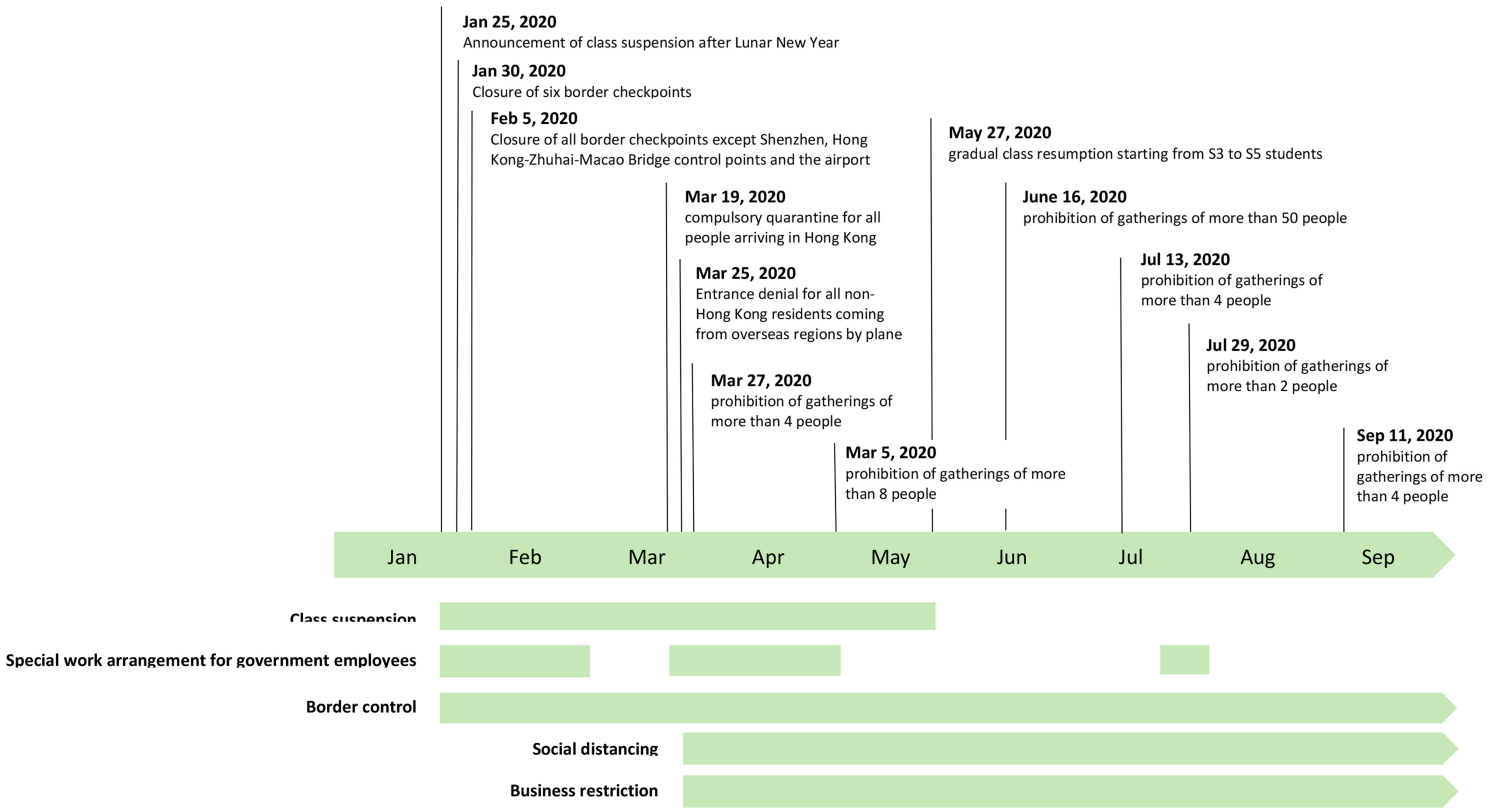
Chronology of major events of COVID-19 control in Hong Kong.

To achieve these, we administered a population-based telephone survey in May 2020 when the control measures for COVID-19 were vigorously implemented in Hong Kong (Figure 1). Hong Kong is one of the models of an information society with a high percentage of Information and Communication Technology penetration among its citizens. According to the report of the Census and Statistics Department in 2020, the percentages of households with a personal computer at home, households with access to the Internet, Internet use, and mobile phone use in Hong Kong were 77.6, 94.1, 91.5, and 91.7%, respectively (Census and Statistics Department, 2020). Thus, it is important to understand PTU and its factors in this large population to estimate healthcare needs and inform future intervention design.

## Materials and Methods

### Participants and Procedure

A population-based telephone survey was conducted in Hong Kong. The interviewers were trained by the principal investigator, had at least 6-months interviewing experience, and were supervised on-site by a senior project coordinator. Telephone numbers were randomly drawn from the 2020 Hong Kong residential telephone directory by computer. Another three sets of numbers were then generated using the randomization of the last two digitals to recruit the unlisted numbers. Eligible household members whose day-month of birth was closest to the survey date were invited to join the study. Two follow-up calls were conducted for unanswered calls before a telephone number was considered invalid. The interviewers briefly introduced the aim of the survey and then asked for verbal informed consent from the potential participants. The anonymous interview took 10–15 min. No incentive was given. The study procedures were carried out in accordance with the Declaration of Helsinki. The study was approved by the Survey and Behavioral Research Ethics Committee of the corresponding author’s affiliating university (Ref.# SBRE-19-645).

Inclusion criteria of the participants included as: (1) Chinese speaking, (2) 18 years old or above, (3) Hong Kong residents (holders of Hong Kong identity cards), and (4) having any experience in using social media (e.g., Facebook, Twitter, WhatsApp, and WeChat) or in playing Internet games in prior 12 months (i.e., recent social media users or Internet gamers). Of the 1882 individuals reached, 1,070 agreed to participate in this study; 658 recent social media users/177 Internet gamers were identified and completed the interview.

### Measures

The symptoms of SMA among social media users were measured by the 6-item Bergen Social Media Addiction Scale (BSMAS; Andreassen et al., 2016). The scale was developed based on the six core components of addictive behaviors, including cognitive salience, tolerance, mood modification, difficulty in regulating use, withdrawal, and interference with role performance. The items are rated on Likert scales, ranging between 1 (very rarely) and 5 (very often). A higher sum score in the BSMAS indicates a greater level of SMA symptoms. The Chinese version was demonstrated to have good psychometric properties (Leung et al., 2020). The scale received good reliability in social media users (Cronbach’s alpha = 0.80) in the current study.

The symptoms of IGD among Internet gamers were measured by the 9-item DSM-5 IGD symptoms checklist. It is a short, user-friendly, and self-reported measure assessing IGD symptoms of preoccupation, tolerance, withdrawal, unsuccessful attempts to limit gaming, deception or lies about gaming, loss of interest in other activities, use despite knowledge of harm, use for escape or relief of negative mood, and harm based on DSM-5 criteria (American Psychiatric Association, 2013). Response options include “no” (0) and “yes” (1). A higher sum score indicates a higher level of IGD symptoms. A score of 5 is taken as the cutoff point for defining IGD. The Chinese version was found to have good psychometric properties in Hong Kong adults and widely used in our previous studies (Ko et al., 2014; Sigerson et al., 2017; Yang et al., 2020; Yu et al., 2020; Yang et al., 2021). The scale reliability was acceptable in Internet gamers (Cronbach’s alpha = 0.75).

Post-traumatic stress among due to COVID-19 both social media users and Internet gamers was assessed by the 8-item Post-Traumatic Stress Disorder Scale (PTSD-8; Hansen et al., 2010). The items correspond to the DSM-IV criteria for PTSD and were modified to ask participants’ responses in the context of COVID-19 in this study. The items are rated on Likert scales from 1 “not at all” to 4 “all the time.” Scores from individual items are summed to provide an overall score, and a higher overall score suggests greater post-traumatic stress due to COVID-19. The Chinese version was obtained by using the translation and back-translation method by two psychologists of the research panel. The scale reliability was acceptable in social media users (Cronbach’s alpha = 0.74) and Internet gamers (Cronbach’s alpha = 0.78).

Boredom during COVID-19 among both social media users and Internet gamers was measured by three items of the Multidimensional State Boredom Scale (Hunter et al., 2015). The items (i.e., “I feel bored during COVID-19,” “I am easily distracted during COVID-19,” and “Time is passing by slower than usual during COVID-19”) are rated on Likert scales (1 = strongly disagree to 5 = strongly agree). A higher sum score indicates a higher level of boredom during COVID-19. The Chinese version has been used in the previous studies (Liu et al., 2013). The scale reliability was good in social media users (Cronbach’s alpha = 0.86) and Internet gamers (Cronbach’s alpha = 0.83).

Loneliness during COVID-19 among both social media users and Internet gamers was assessed by the Chinese version of the 6-item De Jong Gierveld Loneliness Scale (Leung et al., 2008). It includes two subscales of emotional loneliness and social loneliness (Leung et al., 2008). Response options include “no,” “more or less,” and “yes.” A higher sum score suggests a higher level of loneliness during COVID-19. The Cronbach’s alpha was relatively low but acceptable (Cronbach’s alpha = 0.67 in social media users and 0.68 in Internet gamers).

Socio-demographic information of sex, age, current marital status, education level, income, health status, and quarantine status [i.e., whether one had been subjected to compulsory quarantine at designated places (home, hotel, or other accommodation) under government order for COVID-19 infection control] was collected. Consistent with the previous research, screen time before and during COVID-19 was measured by asking the participants how long did they spend on social media use and Internet gaming daily before and during the COVID-19 isolation, respectively (Cellini et al., 2020). The changes in screen time were obtained by subtracting the screen time score before COVID-19 from the screen time score during COVID-19 (Cellini et al., 2020).

### Statistical Analyses

Descriptive statistics were computed for all variables among users of social media and Internet games. The levels of SMA and IGD symptoms by socio-demographic status were compared by either *t*-test or ANOVA. Simple linear regression analyses using raw data were conducted to test the associations between the key psychosocial variables and SMA/IGD symptoms. Standardized regression coefficients (*β*) and 95% confidence interval (CI) were reported. SPSS version 21.0. was used for these analyses. Mediation analyses were conducted by PROCESS (Hayes, 2017). Bootstrapping based on 5,000 bootstrap samples was performed to test mediation effects. The significant socio-demographic variables of SMA/IGD symptoms were adjusted for in mediation analyses. The level of statistical significance was 0.05.

## Results

### Socio-Demographic Characteristics of the Participants

Socio-demographic characteristics of the social media users and Internet game users were presented in Table 1. More than half of the social media users were female (63.2%), aged 36–65 years (64.4%), and were currently married/cohabiting with a partner (67.5%). About half of them had a secondary school education level or below (53.9%), and 57.3% had monthly household income of 30,000 HKD or below (57.3%). Most of them had no chronic disease (78%) or mandatory quarantine experience (98%). Half of the Internet gamers were female (53%); 40% aged 36–65; and 47% were single. Less than half of them had a secondary school education level or below (45.2%), and three-fifths had monthly household income of 30,000 HKD or below (61.0%). Most of them had no chronic disease (81.4%) or mandatory quarantine experience (96.6%).

**Table 1:** Socio-demographic characteristics of the participants.

Socio-demographic characteristics	Social media users*n*(%)	Internet game users*n*(%)
**Sex**		
Male	242(36.8)	84(47.5)
Female	416(63.2)	93(52.5)
**Age group**		
18–25	72(10.9)	46(26.0)
26–35	41(6.2)	25(14.1)
36–45	98(14.9)	28(15.8)
46–55	131(19.9)	18(10.2)
56–65	34(19.2)	
>65	110(16.7)	21(11.9)
Refused to answer	11(1.7)	5(2.8)
**Current marital status**		
Single	176(26.7)	84(47.5)
Cohabiting/married	444(67.5)	82(46.3)
Separated/divorced/widowed	24(3.6)	7(4.0)
Refused to answer/missing value	14(2.1)	4(2.3)
**Educational level**		
Primary school or below	74(11.2)	16(9.0)
Secondary school	281(42.7)	64(36.2)
College or above	281(42.7)	92(52.0)
Refused to answer	22(3.3)	5(2.8)
**Monthly household income (HKD)**		
20,000 or below	265(40.3)	74(41.8)
20,001–30,000	112(17.0)	34(19.2)
30,001–50,000	100(15.2)	28(15.8)
>50,000	78(11.9)	15(8.5)
Refused to answer/missing value	103(15.7)	26(14.7)
**Having chronic diseases (e.g., hypertension, diabetes, and cancer)**		
Yes	145(22.0)	33(18.6)
No	513(78.0)	144(81.4)
**Subjected to mandatory quarantine**		
Yes	13(2.0)	6(3.4)
No	645(98.0)	171(96.6)

**Figure 2:**
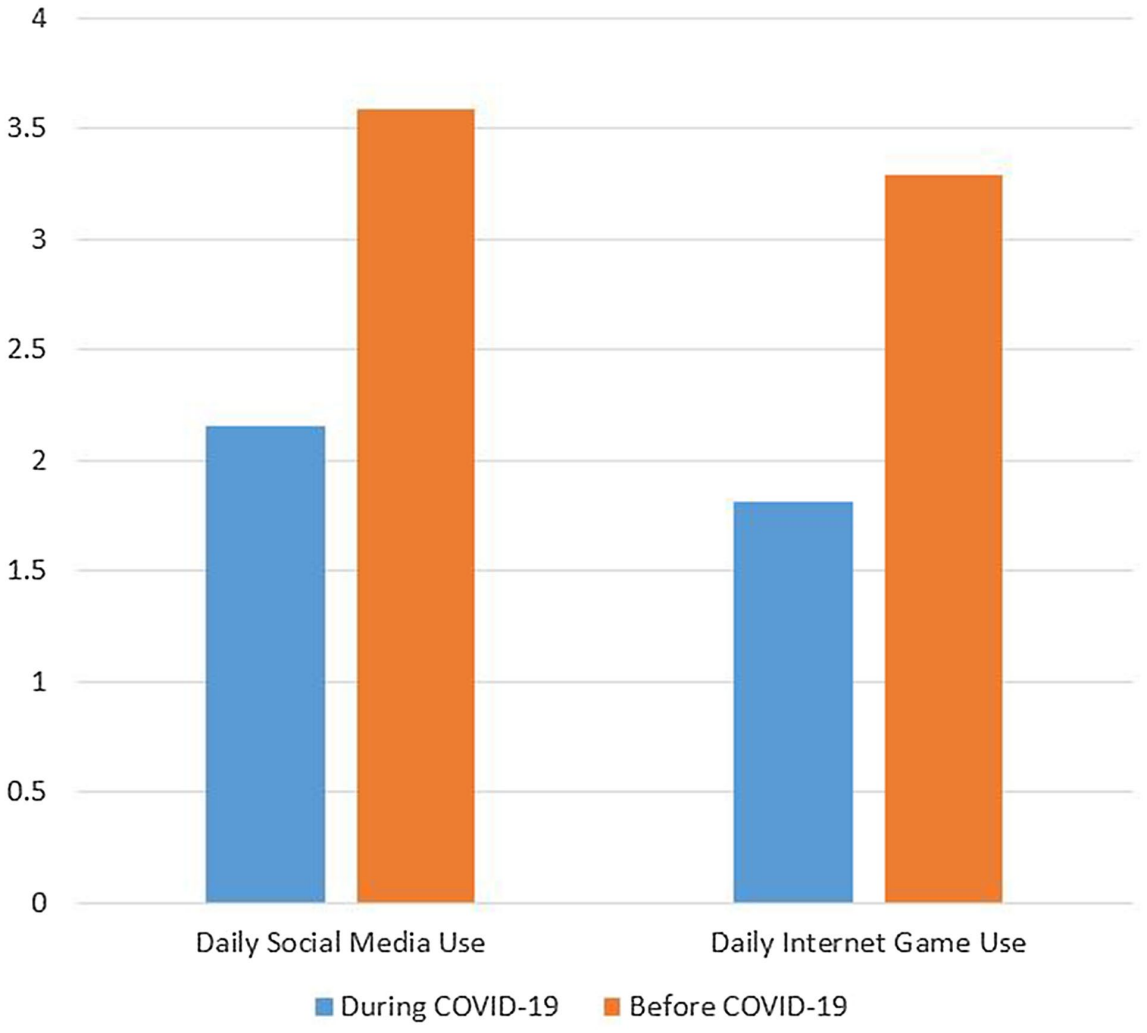
Daily use of social media and Internet game before and during COVID-19 (hours).

As Figure 2 showed, daily time use of social media during COVID-19 significantly increased compared to pre-COVID-19 (3.59 vs. 2.16 h; *t* = 33.28, *p* < 0.001). There was a 66.2% increase in time use of social media. Similarly, daily time use of Internet gaming during COVID-19 significantly increased compared to pre-COVID-19 (3.29 vs. 1.81 h; *t* = 19.59, *p* < 0.001). An 81.8% increase in time use of Internet gaming was identified. The estimated IGD prevalence rate in the gamers based on the sample weighted to the age distribution and gender ratio of the Hong Kong population was 9.7%. This result was not tabulated.

### Endorsement Rate on Each Criterion for SMA and IGD

**Figure 3:**
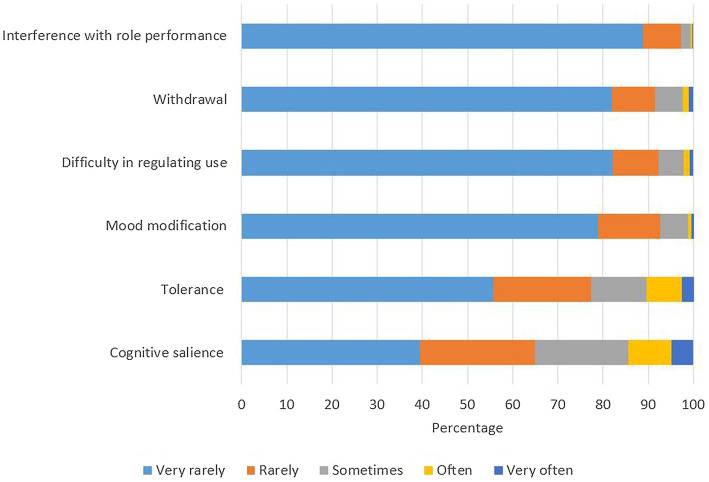
Endorsement rate of each item of social media addiction scale among social media users (*n* = 658).

The most commonly endorsed (sometimes/often/very often) criteria of SMA included cognitive salience (35.0%) and tolerance (22.7%; Figure 3). Few social media users endorsed the criterion of interference with role performance (2.7%).

**Figure 4:**
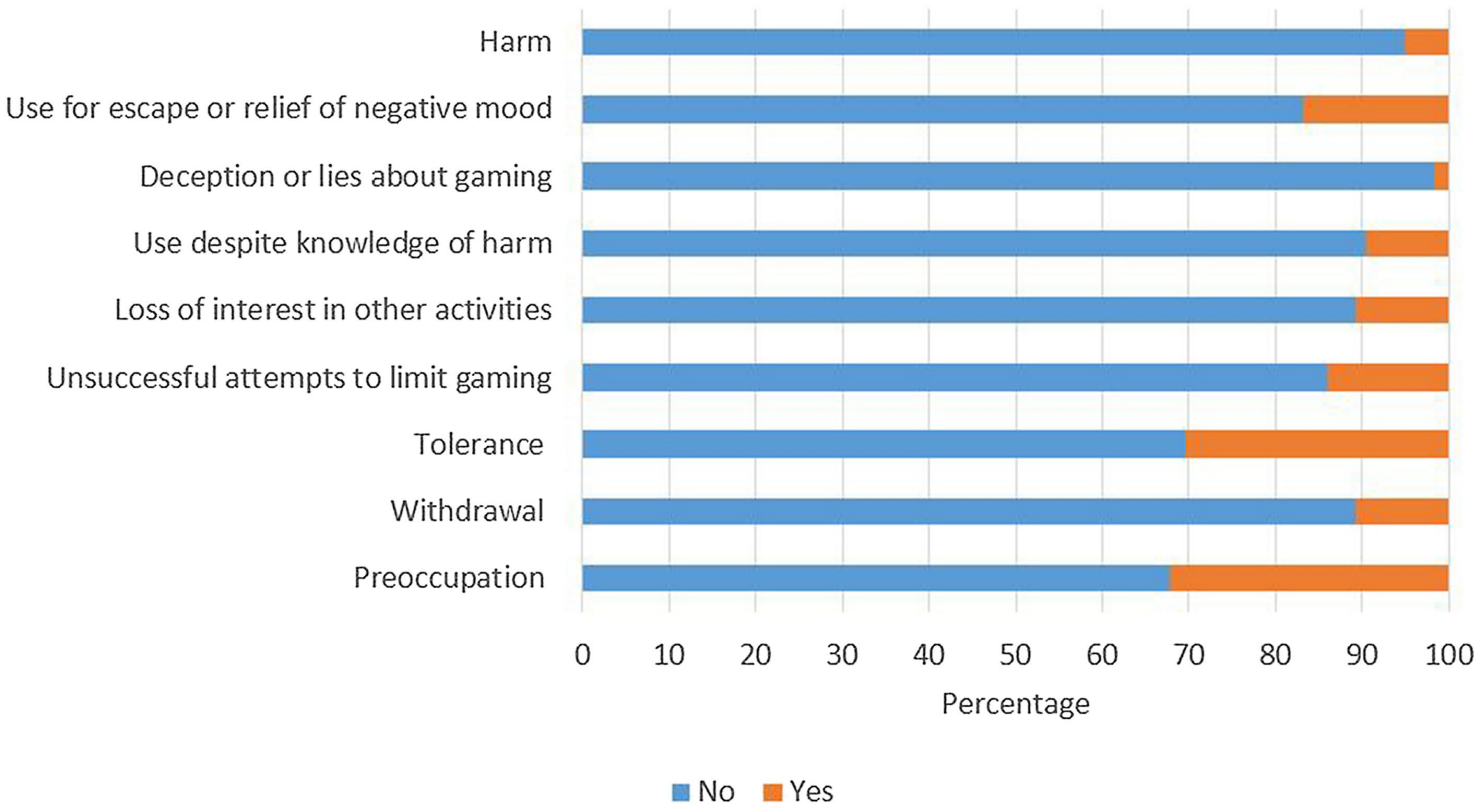
Endorsement rate of each item of Internet gaming disorder scale among Internet gamers (*n* = 177).

Similarly, one-third of the Internet gamers endorsed the dimensions of preoccupation (32.2%) and tolerance (30.5%; Figure 4). 16.9% of them endorsed that they played Internet games for escape or relief of negative mood during COVID-19. Only 1.7% of the Internet gamers endorsed the criterion of deception or lies about gaming.

### Socio-Demographic and Psychosocial Correlates of SMA/IGD Symptoms

As Table 2 showed, individuals who were younger or single, or had higher education levels showed higher SMA scores than their counterparts (*p* < 0.001). IGD scores were not significantly different by socio-demographic characteristics. However, further subgroup analyses showed that female gamers aged 18–25 (Mean = 1.94, *SD* = 2.20; proportion of having IGD = 16.7%) and male gamers aged 18–45 showed higher levels of IGD symptoms/proportions of IGD (Mean = 2.08, *SD* = 2.19; proportion of having IGD = 28.6%) than their counterparts, respectively. These results were not tabulated.

**Table 2:** The levels of social media addiction and Internet gaming disorder symptoms by socio-demographic characteristics.

Socio-demographic characteristics	SMA symptoms			IGD symptoms		
	Mean ± *SD*	*F*/*t*(df)	Value of *p*	Mean ± *SD*	*F*/*t*(df)	Value of *p*
**Sex**		0.64	0.52		0.20	0.84
Male	9.10 ± 3.68	(656)		1.35 ± 1.99	(155.67)	
Female	8.91 ± 3.56			1.29 ± 1.54		
**Age group**		12.03	<**0.001**		1.04	0.40
18–25	10.68 ± 4.02	(5,641)		1.50 ± 2.06	(5,166)	
26–35	11.27 ± 3.78			1.29 ± 1.64		
36–45	9.87 ± 3.67			1.86 ± 2.29		
46–55	8.81 ± 3.38			1.14 ± 1.28		
56–65	8.18 ± 3.45			1.04 ± 1.37		
>65	8.03 ± 2.91			0.83 ± 1.20		
**Current marital status**		21.89	<**0.001**		1.13	0.32
Single	10.46 ± 3.81	(2,641)		1.54 ± 2.00	(2,170)	
Cohabiting/married	8.42 ± 3.33			1.15 ± 1.53		
Separated/divorced/widowed	0.33 ± 3.99			1.00 ± 1.29		
**Educational level**		21.75	<**0.001**		0.82	0.44
Primary school or below	7.68 ± 3.09	(2,633)		1.19 ± 1.11	(2,169)	
Secondary school	8.37 ± 3.02			1.16 ± 1.56		
College or above	10.03 ± 4.05			1.51 ± 2.00		
**Household income (HKD)**		1.60	0.17		2.22	0.07
20,000 or below	8.71 ± 3.59	(3,550)		1.28 ± 1.79	(3,146)	
20,001–30,000	9.38 ± 3.66			1.71 ± 1.96		
30,001–50,000 > 50,000	9.25 ± 3.409.12 ± 3.36			1.61 ± 2.170.53 ± 0.74		
**Having chronic diseases (e.g., hypertension, diabetes, and cancer)**		−0.61(656)	0.54		0.17(175)	0.87
Yes	8.82 ± 3.47			1.36 ± 1.65		
No	9.03 ± 3.64			1.31 ± 1.79		
**Subjected to mandatory quarantine**		1.81(656)	0.07		−0.68(175)	0.50
Yes	10.77 ± 4.66			0.83 ± 1.33		
No	8.95 ± 3.57			1.33 ± 1.78		

The bold values indicate statistical significance at *p* < 0.001.

Simple regression analyses showed that time spent on social media (*β* = 0.37, 95% = 0.53, 0.78), post-traumatic stress (*β* = 0.27, 95%CI = 0.18, 0.32), boredom (*β* = 0.21, 95%CI = 0.15, 0.31), and emotional loneliness (*β* = 0.17, 95%CI = 0.26, 0.66) were significantly and positively associated with SMA symptoms. Time spent on Internet gaming (*β* = 0.43, 95%CI = 0.31, 0.60), boredom (*β* = 0.27, 95%CI = 0.06, 0.21), and emotional loneliness (*β* = 0.15, 95%CI = 0.004, 0.36) was significantly and positively associated with IGD symptoms. These results were not tabulated.

### Mediation Testing

Mediation analyses revealed that time spent on social media was significantly associated with SMA symptoms directly and indirectly through boredom (*β* = 0.03, 95%CI = 0.01, 0.06) and social loneliness (*β* = −0.01, 95%CI = −0.02, −0.001), respectively (Table 3). Time spent on Internet gaming was significantly associated IGD symptoms directly and indirectly through boredom (*β* = 0.04, 95%CI = 0.003, 0.09). Hence, the partial mediation effects of boredom and social loneliness on the relationships between screen time and SMA/IGD symptoms were demonstrated.

**Table 3:** Direct and indirect effects of screen time on SMA/IGD symptoms.

	*β* of direct effect (95% CI)	*β* of indirect effect (95% CI)
**Mediation model of SMA symptoms**		
Model 1: Time use of social media → SMA *via* post-traumatic stress	0.35^***^ (0.52, 0.76)	0.01 (−0.01, 03)
Model 2: Time use of social media → SMA *via* boredom	0.33^***^ (0.46, 0.73)	0.03^*^ (0.01, 0.06)
Model 3: Time use of social media → SMA *via* emotional loneliness	0.36^***^ (0.52, 77)	0.01 (−0.01, 0.02)
Model 4: Time use of social media → SMA *via* social loneliness	0.38^***^ (0.54, 0.80)	−0.01^*^ (−0.02, −0.001)
**Mediation model of IGD symptoms**		
Model 1: Time use of Internet games → IGD *via* PTSD	0.43^***^ (0.31, 0.60)	0.001 (−0.01, 0.01)
Model 2: Time use of Internet games → IGD *via* boredom	0.39^***^ (0.27, 0.56)	0.04^*^ (0.003, 0.09)
Model 3: Time use of Internet games → IGD *via* emotional loneliness	0.44^***^ (0.32, 0.61)	−0.02 (−0.05, 0.01)
Model 4: Time use of Internet games → IGD *via* social loneliness	0.43^***^ (0.31, 0.60)	−0.001 (−0.03, 01)

Significant socio-demographic variables of SMA symptoms (age, current marital status, and educational level) were adjusted for:

*** *p* < 0.001;

* *p* < 0.05.

## Discussion

To our best knowledge, this is the first study estimating the prevalence of IGD in a population-based sample during the COVID-19 isolation. It is also the first study on the roles of technology use and the potential mechanisms in behavioral health (SMA and IGD) in this particular period. In addition, it represents the first study extending the application of the COR theory to understand the development of SMA and IGD. The findings highlight concerns regarding the increased screen time and IGD prevalence and suggest potential psychosocial mechanisms between screen time and SMA/IGD symptoms. The hypotheses were generally supported by the data.

Our results based on a retrospective reporting approach suggest a significant increase in time spent on social media use and Internet gaming in the general population during COVID-19 compared to pre-COVID-19. Other studies in young adults in other countries/regions used the same approach and reported similar findings (Cellini et al., 2020; Sun et al., 2020). The significant change in screen time may reflect the fact that people have relied on digital platforms for information, social connection, entertainment, and fulfillment of other basic needs during the COVID-19 isolation (Ko and Yen, 2020).

Although technology use has become inevitable and provided benefits during COVID-19 (Liu and Kim, 2011; Shah et al., 2020; Zhou et al., 2020), it is worth noting that related PTU may have subtly increased. Supporting this argument, we found that the estimated IGD prevalence is almost 10% during COVID-19 which is higher than that of pre-COVID-19 surveys in Hong Kong (8.3%; Wang and Cheng, 2021) and Macao (4.3%) using the same sampling method (i.e., population-based telephone surveys; Wu et al., 2018). It echoes and provides empirical evidence regarding the concern about the elevated risk of IGD due to the COVID-19 isolation and strain in recent commentary papers (Górnicka et al., 2020; King et al., 2020; Ko and Yen, 2020). Whether this growing tendency would remain unchanged or drop when alternative options of entertainment (e.g., outdoor activities) become available should be monitored in follow-up work. Monitoring the change in IGD at different stages of COVID-19 would facilitate researchers and healthcare service providers to better understand the nature of IGD (e.g., stability over time), estimate related healthcare service needs, and conduct health promotion programs for early detection and treatment. Furthermore, our results suggest that some subgroups, such as young females and males, may have a high risk of IGD and deserve particular attention in research and service practice. Especially for young females, their IGD has been highlighted as a hidden and understudied problem. The “gender gaps” in gaming participation and IGD prevalence have been closing in recent years (King and Potenza, 2020); it is very possible that such gaps may be further narrowed during the COVID-19 isolation.

The participants reported a relatively low level of SMA as the mean score was lower than the midpoint of the scale. Since our study is the first attempt investigating SMA in community adults of Hong Kong, pre- and during-COVID-19 comparison is not available. One pre-COVID-19 study was conducted among local college students and reported a higher level of SMA (Mean = 14.58; Leung et al., 2020). However, the sample characteristics and sampling methods were not comparable between the two studies. Furthermore, we found those who were younger, single, or with higher education levels had higher levels of SMA. These findings are consistent with the previous non-COVID-19 studies (e.g., Blackwell et al., 2017). Further stratification based on these risk characteristics and follow-up studies to better understand the changes in PTU at different stages of COVID-19 are warranted.

Among the symptoms/criteria of SMA and IGD, the participants were more likely to endorse the cognitive symptoms (e.g., cognitive salience/preoccupation and tolerance) and less likely to endorse the symptoms related to the negative consequences of social media use/Internet gaming (e.g., interference with role performance/deception or lies about gaming). Such patterns are consistent with those found in the pre-COVID-19 study in adults (Leung et al., 2020). It may be because it often takes time before the negative consequences of these technology uses become salient to a person. Another plausible explanation particularly relevant to COVID-19 is that this pandemic and related control measures have normalized excessive use of digital platforms, and thus, people are less likely to recognize or experience its negative consequences in this particular period. However, the potential increases in the consequence-related symptoms are worth attention if the COVID-19 crisis persists or when it has ended and people start to experience difficulties in re-adaptation. This issue may be more salient among children and adolescents as they are especially vulnerable to PTU at the developmental stage and have failed to follow the routine of going to school or participate in other activities during COVID-19 (Ko and Yen, 2020).

Furthermore, we found strong positive relationships between screen time during COVID-19 and PTU, consistent with the findings in the pre-COVID-19 studies (e.g., Stavropoulos et al., 2019) and studies during COVID-19 (Shrestha et al., 2020; Chen et al., 2021). Although the use of such digital platforms may have the potential to ameliorate the negative impact of social distancing on individuals and facilitate COVID-19 control (Ghebreyesus, 2020; Maden, 2020), prolonged use might increase PTU which is associated with a range of severe mental health problems and may lead to difficulties in re-adaptation after the COVID-19 crisis has passed (King et al., 2020; Ko and Yen, 2020; Sun et al., 2020). In addition, the significant psychosocial correlates of PTU during COVID-19 included perceived post-traumatic stress, state boredom, and emotional loneliness. Consistently, the previous studies also reported that individuals with greater PTSD due to maltreatment during childhood, boredom proneness, leisure boredom, or loneliness tend to be more vulnerable to PTU (e.g., Zhou and Leung, 2012; Boursier et al., 2020). This study is the first attempt to extend the associations between PTSD/boredom and PTU into the context of the COVID-19 crisis. Our finding is also consistent with the recent studies on the relationship between loneliness and PTU during COVID-19 (e.g., Zhou and Leung, 2012; Boursier et al., 2020; T’ng et al., 2020). These findings support the compensatory model of PTU which asserts that the reactions to negative life circumstances (e.g., negative emotions due to COVID-19) might increase excessive Internet use and addictive-like symptoms (Kardefelt-Winther, 2014a). Since COVID-19 may be a temporary stressor, whether such emotional reactions may last after COVID-19 or have a long-term effect on PTU should be investigated in future work.

Moreover, the mediation analyses suggest that technology use may be a source of state boredom and reduce social loneliness which may further affect the levels of PTU. Boredom relief and social network maintenance might be two primary motives for the use of digital platforms during the COVID-19 isolation. However, prolonged use might cause an increase in state boredom, instead of successfully alleviating boredom, which could in turn increase both SMA and IGD. An experimental and non-COVID-19 study also demonstrated that spending over 30 min on social media significantly increased state boredom (e.g., Kardefelt-Winther, 2014b; Barkley and Lepp, 2021). The authors explained that digital technology use may not be as intrinsically rewarding as users anticipate; thus it could not improve state boredom and eventually worsened it (Barkley and Lepp, 2021). Additionally, one plausible explanation that is particularly relevant to COVID-19 is that these digital platforms could facilitate the spread of boredom emotions and emotional contagion among users during the COVID-19 isolation. Experimental studies and qualitative interviews are needed to test this possibility. On the other hand, as hypothesized, prolonged use of social media might maintain or enhance interpersonal resources and social networks, and thus reduce social loneliness during the COVID-19 isolation; in turn, adequate interpersonal resources might facilitate one’s adaptive stress coping and reduce the risk of SMA. The negative mediation effect of social loneliness may highlight the beneficial effect of spending more time on social media during the COVID-19 isolation (Ghebreyesus, 2020; Maden, 2020). It is worth noting that some non-COVID-19 studies have argued that longer time spent on social media did not necessarily reduce loneliness; only limiting social media use to approximately 30 min per day would reduce loneliness (King and Potenza, 2020). It is because the longer time one spends on social media, the more one will be engaging with it passively (as opposed to actively posting content, commenting, etc.; King and Potenza, 2020). However, in our study, the participants spent long time on social media daily during COVID-19 (Mean = 3.59 h), and the longer they used it the less loneliness they perceived. It is possible that this beneficial effect may be particularly applicable to the situation of COVID-19 where a range of social and outdoor activities were restricted and people might actively use social media for social connections and interactions.

### Theoretical and Practical Implications

While the findings are preliminary only and should be treated with caution, they have useful implications for understanding behavioral problems in the digital age and in collective traumatic events. The mediation models in this study extend the COR theory (Hobfoll, 1989) by suggesting that personal resource losses, such as a great state of boredom, and interpersonal resource gains, such as reduced social loneliness, induced by technology use might have ripple effects beyond psychosocial status or mental distress, leading to behavioral disorders and PTU. It should be noted that the two mediators showed relatively small effect sizes and the direct effects remained significant. These partial mediation effects may suggest that screen time could affect PTU through other unmeasured resources. For example, prolonged use of social media/Internet games may increase sleep problems, fatigue, and online social support which are risk or protective factors for PTU (e.g., Sigerson et al., 2017; Alimoradi et al., 2019). Such potential mediators should be tested in future studies to better understand the complex mechanisms between screen time and PTU.

These findings have important practical implications. Although prolonged use of technology may be inevitable during the COVID-19 isolation, the studied psychosocial mediators are modifiable and can be used to guide future interventions to prevent PTU. Problem technology use may be a consequence of the loss of alternative options (e.g., face-to-face interactions and outdoor activities) to ease the negative emotions during the COVID-19 isolation. Mental health professionals are suggested to provide emotional support and advice on adaptive emotion regulation strategies, and formulate alternative and feasible social and recreational activities, all of which could be essential to minimizing the risks of PTU during this pandemic. In addition, public health strategies to enhance digital literacy and strategic and healthy use of social media and Internet games in the general public are in urgent need. As a newly defined disorder, people may know little about IGD and pay little effort to prevent it. Educational programs should be conducted to enhance the awareness of IGD and its adverse consequences. To prevent PTU during COVID-19, general lifestyle (e.g., adequate sleep and physical activity) and Internet-specific recommendations (e.g., balanced screen time, using digital wellbeing apps) have been proposed (World Health Organization, 2020) and should be promoted in the general public. Particular effort should be made to detect and prevent potential SMA and IGD in the high-risk groups, such as younger people and those with greater negative emotions.

### Limitations

The study has several limitations. First, the study was cross-sectional in nature. It is plausible that people with higher levels of SMA and IGD are more susceptible to psychosocial status during COVID-19. The cognitive symptoms of SMA and IGD were likely to be endorsed by the participants. In addition, the relationship between screen time and psychosocial status can be bi-directional; in other words, long screen time can be both the cause and consequence of negative psychosocial status. Longitudinal studies to monitor the trajectories of screen time, psychosocial status, and PTU are warranted. Such studies will help to better identify individual vulnerabilities and long-term effects of this pandemic on addictive behaviors and mental health. Furthermore, the cross-sectional design with a relatively low response rate might not be able to reflect the accurate picture of the changes in SMA and IGD in response to COVID-19. Second, self-reported answers were recorded. The levels/prevalence of PTU, screen time before and during COVID-19, and psychosocial status might be subjected to social desirability or recall bias. The IGD screening tool was based on DSM-5 criteria, rather than the updated ICD-11 criteria. Future work should validate the findings of IGD using ICD-11 criteria. Third, sampling might exclude those without land-line telephone but only using mobile phone and those who were not at home during the survey period. Also, the younger generation and heavy social media users and Internet gamers might be less likely to be reached by land-line telephone. It might influence the representativeness of the sample. Fourth, the diagnosis or symptom of COVID-19 of the participants may be significant moderators which was not assessed in this study. Finally, this study only focused on time spent on digital technology. We did not investigate other domains (e.g., content and functions) of digital use. For example, connecting with family members vs. virtual friends *via* SNS may have different social and health implications. Future studies should investigate these domains of digital use to better understand its impacts on mental and behavioral health during COVID-19.

## Conclusion

This population-based study highlighted the concern of prolonged use of social media and Internet games during COVID-19 in Hong Kong. The study demonstrated positive relationships between screen time and SMA/IGD symptoms and the partial mediation effects of increased boredom and reduced social loneliness on these relationships. The inconsistent mediation effects highlight the complex role of digital technology use in individual psychosocial wellbeing and behavioral/mental health during traumatic events. Future longitudinal studies are warranted to investigate the changes in the levels/prevalence of SMA and IGD at different stages of COVID-19 and their risk and protective factors. Particular attention should be paid to and prevention effort should be made for the at-risk groups, including those who are younger, single, or have higher education levels. Other types of PTU (e.g., smartphone addiction) may also become a significant public health concern and should be explored in future studies.

## Data Availability Statement

The raw data supporting the conclusions of this article will be made available by the authors, without undue reservation.

## Ethics Statement

The studies involving human participants were reviewed and approved by the Survey and Behavioral Research Ethics Committee of the Chinese University of Hong Kong (Ref.# SBRE-19-645). Written informed consent for participation was not required for this study in accordance with the national legislation and the institutional requirements.

## Author Contributions

XY, BY, and SW conceived the research questions and supervised the project implementation. XY conducted the statistical analysis and drafted the manuscript. SW designed this study and assembled the team of collaborators. EL and DZ gave comments to intellectual content of the manuscript. All authors contributed to the article and approved the submitted version.

## Conflict of Interest

The authors declare that the research was conducted in the absence of any commercial or financial relationships that could be construed as a potential conflict of interest.

## Publisher’s Note

All claims expressed in this article are solely those of the authors and do not necessarily represent those of their affiliated organizations, or those of the publisher, the editors and the reviewers. Any product that may be evaluated in this article, or claim that may be made by its manufacturer, is not guaranteed or endorsed by the publisher.
